# Neuroprotective Effect of Wharton's Jelly-Derived Mesenchymal Stem Cell-Conditioned Medium (WJMSC-CM) on Diabetes-Associated Cognitive Impairment by Improving Oxidative Stress, Neuroinflammation, and Apoptosis

**DOI:** 10.1155/2023/7852394

**Published:** 2023-04-11

**Authors:** Zohre Aghaei, Narges Karbalaei, Mohammad Reza Namavar, Masoud Haghani, Mahboobeh Razmkhah, Mahdi Khorsand Ghaffari, Marzieh Nemati

**Affiliations:** ^1^Department of Physiology, School of Medicine, Shiraz University of Medical Sciences, Shiraz, Iran; ^2^Histomorphometry and Stereology Research Center, Shiraz University of Medical Sciences, Shiraz, Iran; ^3^Department of Anatomy, School of Medicine, Shiraz University of Medical Sciences, Shiraz, Iran; ^4^Department of Tissue Engineering and Applied Cell Sciences, School of Advanced Medical Sciences and Technologies, Shiraz University of Medical Sciences, Shiraz, Iran; ^5^Department of Endocrinology and Metabolism Research Center, Shiraz University of Medical Sciences, Shiraz, Iran

## Abstract

According to strong evidence, diabetes mellitus increases the risk of cognitive impairment. Mesenchymal stem cells have been shown to be potential therapeutic agents for neurological disorders. In the current study, we aimed to examine the effects of Wharton's jelly-derived mesenchymal stem cell-conditioned medium (WJMSC-CM) on learning and memory, oxidative stress, apoptosis, and histological changes in the hippocampus of diabetic rats. Randomly, 35 male Sprague Dawley rats weighing 260–300 g were allocated into five groups: control, diabetes, and three diabetic groups treated with insulin, WJMSC-CM, and DMEM. The injections of insulin (3 U/day, S.C.) and WJMSC-CM (10 mg/week, I.P.) were done for 60 days. The Morris water maze and open field were used to measure cognition and anxiety-like behaviors. Colorimetric assays were used to determine hippocampus glutathione (GSH), malondialdehyde (MDA) levels, and antioxidant enzyme activity. The histopathological evaluation of the hippocampus was performed by Nissl staining. The expression levels of Bax, Bcl-2, BDNF, and TNF-*α* were detected by real-time polymerase chain reaction (RT-PCR). According to our findings, WJMSC-CM significantly reduced and increased blood glucose and insulin levels, respectively. Enhanced cognition and improved anxiety-like behavior were also found in WJMSC-CM-treated diabetic rats. In addition, WJMSC-CM treatment reduced oxidative stress by lowering MDA and elevating GSH and antioxidant enzyme activity. Reduced TNF-*α* and enhanced Bcl-2 gene expression levels and elevated neuronal and nonneuronal (astrocytes and oligodendrocytes) cells were detected in the hippocampus of WJMSC-CM-treated diabetic rats. In conclusion, WJMSC-CM alleviated diabetes-related cognitive impairment by reducing oxidative stress, neuroinflammation, and apoptosis in diabetic rats.

## 1. Introduction

The worldwide prevalence of diabetes mellitus (DM), a chronic metabolic disorder characterized by hyperglycemia, is rising. Numerous studies have demonstrated that DM harms many organs, including the brain. A close relationship between diabetes and cognitive impairment has been shown [1]. According to epidemiological studies, mild-to-moderate cognitive dysfunction, depression, and anxiety are all two to three times more prevalent in diabetic patients than in nondiabetic people [2]. Diabetes-related dementia is thought to be caused by various factors, including insulin insufficiency, insulin resistance, hyperglycemia, vascular abnormalities, reduced synaptic plasticity, impaired insulin signaling, and oxidative stress in the central nervous system (CNS) [2, 3].

Because of its importance in neurodegenerative diseases and its vulnerability to metabolic disturbances, the hippocampus may be one of the most sensitive CNS regions associated with diabetes [4]. It has been established that cognitive deficits in central nervous system diseases are linked to hippocampus neuron loss and synaptic plasticity damage [5]. Furthermore, insulin receptors and insulin-sensitive transporters are abundant in the hippocampus [6], and insulin receptors in the brain are associated with cognitive functions and are crucial in chronic neurodegenerative diseases such as Alzheimer's and Parkinson's diseases [7, 8].

In diabetes, prolonged hyperglycemia causes an increase in free radical formation and accumulation of reactive oxygen species (ROS) and a decrease in antioxidant defense system activity [9]. Cognitive dysfunction has been linked to inflammation, free radical damage to the hippocampus, and metabolic impairment of neuroprotective factors. Studies have revealed that diabetes increases oxidative stress in rats' hippocampus, damaging the nerve cell membrane and changing the mitochondrial membrane permeability, resulting in neuronal loss [10, 11]. Studies have also indicated that increased inflammation and oxidative stress in the hippocampus and cortex of diabetic animals play a critical role in diabetic neuronal apoptosis, neurotransmitter homeostasis disruption, reduced hippocampal synaptic plasticity, and morphological abnormalities of neurons [3, 12, 13]. Moreover, decreased neuronal proliferation, altered astrocytic properties, and abnormal neuronal activities in the hippocampus have been linked to poor cognitive performance and impaired learning in rodents, particularly in memory tasks [14, 15]. Therefore, inhibiting the aforementioned pathogenetic processes is the key to treating cognitive impairment.

Mesenchymal stem cells (MSCs) have been proven to be capable of tissue repair and treatment of many diseases due to their multipotent differentiation competency and ability to release trophic chemicals that have a beneficial effect on injured tissue [16]. MSCs are also the subject of extensive studies as a potential treatment for neurodegenerative diseases like Parkinson's disease, Alzheimer's disease, Huntington's disease, amyotrophic lateral sclerosis, and multiple sclerosis [17, 18]. Among MSCs, Wharton's jelly-derived MSCs (WJMSCs) from the umbilical cord are the most readily available, have low immunogenicity and proliferative abilities, and can be expanded in vitro. Numerous studies have demonstrated that WJMSCs are potentially promising candidates for cell therapy and can improve neurological function in animal models of traumatic brain damage, ischemic stroke, neonatal hypoxia-ischemia, and Alzheimer's disease [19–21].

MSC-based therapy is a promising treatment strategy in diabetic neuropathy; however, direct transplantation of MSCs to target tissues remains challenging due to poor cell engraftment and survival. Therefore, the application of cell-free products based on MSC-derived secretory factors is considered an advantageous alternative, reducing immune compatibility problems. The MSCs secrete various growth-promoting factors and cytokines which can be found in MSCs cultured medium known as conditioned medium (CM) [22–24]. The MSC-CM exhibited similar effects to MSCs, suggesting that soluble compounds derived from MSCs may be involved. The MSC-CM contains bioactive factors including exosomes and soluble factors such as neurotrophic factors, angiogenic factors, immunosuppressive factors, and other regenerative agents [25–27]. Exosomes are extracellular vesicles that can be secreted by various types of mesenchymal stem cells including human umbilical cord-derived mesenchymal stem cells [28]. They are one component of MSC-generated conditioned medium (CM) and contain numerous biologically active genomic and nongenomic molecules, including DNA, miRNAs, various proteins, enzymes, and lipids, which can influence the biological activity of cells via paracrine or autocrine pathways. It has been indicated that MSC-conditioned medium or its components mediate some biological actions of MSCs [27, 28]. Several studies found that these bioactive factors are capable of repairing tissue in a variety of tissue/organ damage conditions [26, 29]. Due to high levels of nerve growth factor (NGF), brain-derived neurotrophic factor (BDNF), vascular endothelial growth factor (VEGF), and other anti-inflammatory and neuroprotective factors, Wharton's jelly-derived mesenchymal stem cell-conditioned medium (WJMSC-CM) have shown tissue regenerative, anti-inflammatory, antioxidative, and protective effects [30]. Therefore, the current study is aimed at investigating the potential therapeutic effects of WJMSC-CM treatment on learning and memory, hippocampal inflammatory, and stress oxidative markers as well as histology of the hippocampus in STZ-induced diabetic rats.

## 2. Materials and Methods

### 2.1. Animals and Experimental Design

35 adult male Sprague Dawley rats weighing 260–300 g were used in this study. The animals were housed in an environment with a 12-hour light-dark cycle, a controlled temperature (25 ± 1°C), and free access to food and water. The Animal Ethics Committee of the Shiraz University of Medical Sciences assessed and approved all animal experimentation procedures (IR.SUMS.REC.1399.1125).

The rats were randomly distributed into the five experimental groups: control (Con), streptozotocin- (STZ-) induced diabetic rats (Dia), diabetic rats received intraperitoneal injections of 10 mg/day WJMSC-conditioned medium (Dia + CM) or DMEM (Dia + DMEM) once a week for 60 days, diabetic rats received a daily subcutaneous injection of insulin (3 U/day) for 60 days (Dia + INS). The weight of the animals was measured weekly.

**Figure 1:**
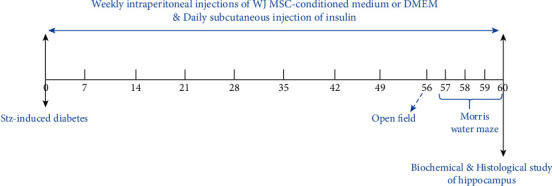
An illustration of the experimental protocol for rats receiving insulin, WJMSC-CM, or vehicle.

After 8 weeks of the start of the experiment, behavioral tasks were performed on the five groups. The open field test (OFT) was performed on the 56^th^, and on days 57-60 of the experiment, the training sessions and probe trials of the Morris water maze (MWM) were done. At the end of the experiment, animals were deeply anesthetized with ketamine (80 mg/kg) and xylazine (10 mg/kg). Blood samples were collected from the heart for the determination of serum levels of fasting glucose and insulin. Then, animals were killed under deep anesthesia, and their brains were removed after decapitation. The right hippocampus was separated and kept at -80°C until processing in the real-time PCR investigation and determination of stress oxidative marker, and the left hippocampus was dissected for stereological assessment.  Figure 1 shows an illustration of the experimental design for rats receiving insulin, WJMSC-CM, or vehicle.

### 2.2. Induction of Experimental Diabetes Model in Rats

A single intraperitoneal injection of 50 mg/kg streptozotocin (diluted in 0.1 M sodium citrate buffer, pH = 4.5) was used to induce diabetes. 72 hours after streptozotocin injection, the hyperglycemia in rats was validated by measuring the fasting blood glucose concentration in tail vein blood with a glucometer. Only diabetic rats with fasting glucose levels greater than 250 mg/dL were examined for this investigation.

### 2.3. Mesenchymal Stem Cell (MSC) Extraction from Wharton's Jelly

After written informed permission and Institutional Ethical Board approval, discarded human umbilical cord (UC) samples were taken at random from healthy full-term neonates delivered in Shiraz University hospital. The UC was transported to the lab within 24 hours under completely sterile conditions. The samples were immediately processed, and delicate Wharton's jelly (WJ) tissue was removed from the amniotic layer and cut into small fragments after three washes in PBS and vessel removal. By slicing and then digesting tissue with collagenase and hyaluronidase enzymes, Wharton's jelly mesenchymal stem cells were extracted. Isolated cells were then cultured in T-25 culture flasks with Dulbecco's Modified Eagle Medium (DMEM) containing 15% fetal bovine serum (FBS) and 1% penicillin/streptomycin (P/S) at 37°C with twice-weekly media changes [31]. It should be mentioned that in research done by our colleagues, such cells were identified as mesenchymal stem cells by the recognition of specific cell surface markers on these cells [32].

### 2.4. Preparation of Freeze-Dried WJMSCs–CM

Wharton's jelly-derived mesenchymal stem cells (WJMSCs) from the fourth passage were used for the conditioned media collection. When the cells were about 80% confluent, the supernatant was removed and the attached cells were washed with PBS. After that, the cells were cultured for 48 hours in serum-free DMEM. Following that, the obtained conditioned media was centrifuged at 1800 rpm for 20 minutes at 4°C to remove cell debris before being filtered through a 0.22 *μ*m syringe filter [33]. Then, supernatants from the WJMSCs were collected and processed into lyophilized powder in a freeze-dryer (Christ Alpha1-2 LD Plus, Germany) and concentrated to 50-fold of the original concentration. The protein of lyophilized WJMSC-CM was quantified by commercial Thermo Scientific BCA Protein Assay Kit (Rockford, IL) according to manufacturer's instructions and then stored at -80°C until further use. The final protein concentration of lyophilized conditioned media that used in this study was 10 mg/mL [34].

### 2.5. Serum Parameter Analyses

Insulin and glucose serum concentrations were evaluated by ELISA methods (Insulin, Mercodia, Uppsala, Sweden) and glucose oxidase procedure (Pars Azmoon Co., Tehran, Iran), respectively. The intra-assay coefficients of variation for insulin and glucose were 9.35% and 5.82%, respectively. The interassay coefficients of variation for insulin and glucose were 10.67% and 7.25%, respectively. All measurements were carried out in duplicate.

### 2.6. Behavioral Assessment

After 8 weeks of the experiment, the following behavioral tests were conducted on the rats. The animals were evaluated blindly by an investigator, and data were obtained using a video image motion analyzer.

#### 2.6.1. Morris Water Maze Test

On days 57 to 60 of the experiment (4 days), the animals were exposed to the Morris water maze test (MWM) to assess spatial learning and memory. The task was assigned between 10 : 00 and 13 : 00. The device consisted of a circular pool (140 cm width and 45 cm height) filled with water (23 ± 2°C). Data was obtained automatically using a video image motion analyzer [Ethovision, Noldus Information Technology, Netherlands]. In the center of one quadrant of the pool, a platform (15 cm width by 35 cm height) was placed 1.5 cm above (visible) or below (submerged) the water's surface. The test was done on day 57 of visible platform training to acclimatize rats to the pool and assess their swimming ability and vision. During hidden platform training (days 57–60), the tasks were performed to evaluate the level of learning. Each day, rats completed four trials with 30-minute intervals. At random, rats were put into the water in one of four quadrants while facing the wall of the maze. For acquisition, the location of the hidden platform remained fixed, and rats were given 60 seconds to swim to it.

The platform was removed on day 60 (after the last training trial), and the probe trial was performed to measure the extent of memory. Rats were permitted to swim for 60 seconds, and the distance and time spent in the target quadrant, where the platform had previously been placed, were measured to determine the retention of spatial memory [35].

#### 2.6.2. Open Field Test (OFT)

The open field is used to measure anxiety and exploratory activity as well as locomotion. The open field apparatus was constructed of a rectangular plexiglass box (90 × 90 with 45 cm height) with a floor divided into sixteen equally sized squares. Four squares were designated as the center, while twelve squares were designated as the periphery. On day 56 of the experiment, the locomotion and anxiety-like behaviors were assessed with the number of grooming, the total distance traveled, and time spent in the central and peripheral area for 5 minutes. The video recordings were analyzed offline using EthoVision (Noldus Information Technology, Netherlands) video tracking software.

### 2.7. Evaluation of Hippocampal Oxidative Stress Markers

Following behavioral tests, rats were deeply anesthetized with I.P. injections of ketamine (80 mg/kg) and xylazine (10 mg/kg) and sacrificed through decapitation. After removing the brain, hippocampal tissue was dissected and then homogenized in cold PBS (pH = 7.4). Samples were centrifuged (10,000 g, 4°C), and supernatants were collected and kept at -80°C until further analysis.

The malondialdehyde (MDA) level, as lipid peroxidation index, was assessed by using the thiobarbituric acid reactive substance method (TBARS) [36]. Superoxide dismutase (SOD) and glutathione peroxidase (GPx) activities as well as glutathione (GSH) concentration were quantified by commercially available assay kits (Kiazist Life Sciences, Iran) using the colorimetric method. To quantify the amount of protein in tissue, the BCA (bicinchoninic acid) Protein Quantification Kit (Parstous, Iran) was used.

### 2.8. Total RNA Extraction and Real-Time PCR

The total RNA from rat hippocampal samples was extracted according to the manufacturer's protocol. In brief, hippocampal tissues were homogenized in Trizole reagent (Standard RNX Plus, SinaClon, Sinc Biotech, Iran) and then mixed with chloroform and centrifuged at 13000 rpm at 4°C for 15 min. The upper supernatant was mixed with isopropanol in a 1 : 1 ratio and centrifuged at 4°C for 15 minutes at 13000 rpm. After completely removing the supernatant, the pellets were washed twice with 70% ethanol and then air-dried. After that, the mRNA pellets were dissolved in 15 *μ*L RNase-free water. A NanoDrop ND-1000 spectrophotometer (A260/A280 > 1.8 and A260/A230 > 1.6; NanoDrop Technologies Inc., Wilmington, DE, USA) was used to assess the concentration and purity of mRNA. By using a cDNA Synthesis Kit (Fermentas, Berlin, Germany), the whole RNA was reversely transcripted to cDNA according to the manufacturer's instructions.

Next, gene expressions of Bcl-2-associated X protein (Bax), B-cell lymphoma 2 (Bcl-2), brain-derived neurotrophic factor (BDNF), and tumor necrosis factor-alpha (TNF-ɑ) in the hippocampus were quantified by real-time PCR on StepOne Real-Time PCR system, using SYBR Green High ROX Master Mix (Amplicon, Brighton, UK) according to manufacturer's instructions. The expression of the cellular housekeeping gene, glyceraldehyde 3-phosphate dehydrogenase (GAPDH), was used to normalize the data of target genes. The relative expression level of genes was calculated by the 2^−ΔΔCt^.  Table 1 lists the primer sequences utilized in this study.

### 2.9. Histological and Stereological Studies

#### 2.9.1. Tissue Processing and Cresyl Violet Staining (Nissl Staining)

After inducing deep anesthesia the rats with intraperitoneal injections of ketamine (80 mg/kg) and xylazine (10 mg/kg) and decapitation, the brains were dissected into left and right hemispheres using a razor blade. The left hemispheres were fixed with 4% paraformaldehyde in a phosphate buffer solution before being transferred to 30% sucrose solution in PBS at 4°C. The samples were sectioned after being embedded in cryomolds in OCT. Fifty *μ*m-thick coronal sections of the hippocampus were cut sequentially by cryostat (SLEE, Frankfurt, Germany) and stored in a cryoprotectant solution at -20°C until used for cresyl violet staining. Using the uniform systematic random sampling method, every 7th section (with 350 *μ*m intervals) was mounted on gelatin-coated slides and kept at room temperature for one night. Following that, sections were stained with cresyl violet to estimate volume and count neurons, glial cells, and endothelial cells [37].

#### 2.9.2. Estimation of the Volume

Through a stereo microscope (Nikon, SM Z745 T, Japan), the image of each cresyl violet stained brain section was transmitted to the monitor. Estimation of the hippocampus volume was accomplished using point counting based on Cavalier's principle by stereological software (Stereo Lite, SUMS, Shiraz, Iran). The following formula was used for volume estimating:

(1) $$\begin{array}{c} V=\left(\sum P\right)\times \left(\frac{a}{p}\right)\times T, \end{array}$$

where ∑*P* is the total point counts (the total number of points hitting the structure), *a*/*p* represents the area per point, and *T* is the section interval.

#### 2.9.3. Estimation of Numerical Density and the Total Number of Neurons, Glial Cell Types, and Endothelial Cells

The optical disector method was used to estimate the numerical density and the total number of cells (neurons, different types of glial cells, and endothelial cells) in the hippocampus. In brief, the optical disector setting included an Eclipse microscope (E200, Nikon, Tokyo, Japan) with a high numerical aperture (*NA* = 1.4) × 60 oil immersion objective, which was connected to a video camera. It transfers the microscopic image to a monitor, and an electronic microcator equipped with a digital readout (MT12, Heidenhain, Traunreut, Germany) for evaluating the movements in the *Z* direction with 0.5 *μ*m precision. A computer-generated unbiased counting frame was superimposed on the screen, using a stereology software system (StereoLite, SUMS, Shiraz, Iran). The mean density of cells in each region was determined as follows:

(2) $$\begin{array}{c} N_{v}=\frac{\sum Q}{\left[\sum P\times a\left(f\right)\times h\right]}, \end{array}$$

where *N*_*v*_ is numerical density, ∑*Q* is the total number of counted neurons or glial cells within the sampling volume, ∑ is the total counted disectors, *a*(*f*) is the area of the frame, and *h* is the height of disector.

The total number of cells was assessed by multiplying numerical density (*N*_*v*_) and volume of area (*V*) [37].

#### 2.9.4. Cytological Features of Neurons, Glial Cell Types, and Endothelial Cells in Rat Hippocampus

**Figure 2:**
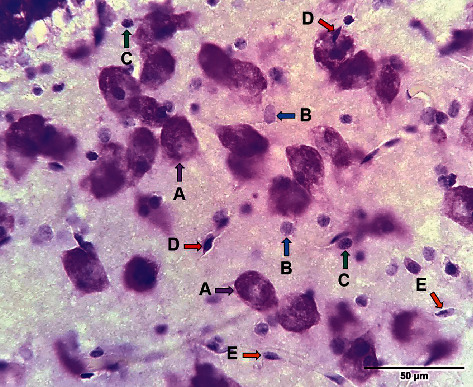
Neurons, glial cell types, and endothelial cells in a cresyl violet stained section of rat hippocampus. Neurons (A, purple arrows), astrocytes (B, blue arrows), oligodendrocytes (C, green arrows), microglia (D, red arrows), and endothelial cells (E, orange arrows).

Although detecting types of glial cells will be best by immunohistochemistry, however, based on a previous study [38], it is possible to differentiate these cells by Cresyl Violet Staining (Nissl Staining).  Figure 2 demonstrated neurons (A), different types of glial cells (B-D), and endothelial cells (E) in a cresyl violet-stained section of rat hippocampus.

### 2.10. Statistical Analysis

The data are calculated using GraphPad Prism (version 8.4.3). We used two-way repeated-measures ANOVA to evaluate of swimming speed, traveled distance, and time in 4 days of the MWM test. One-way ANOVA with Tukey HSD multiple comparison test as post hoc test was used for the comparison of body weight, biochemical data, swimming in the correct quadrant, OFT, and stereological analysis. Results are expressed as mean ± standard error of the mean (SEM). A probability level of *P* < 0.05 was considered as a criterion for statistical significance.

## 3. Results

### 3.1. Body Weight and Serum Glucose and Insulin Levels

**Figure 3:**
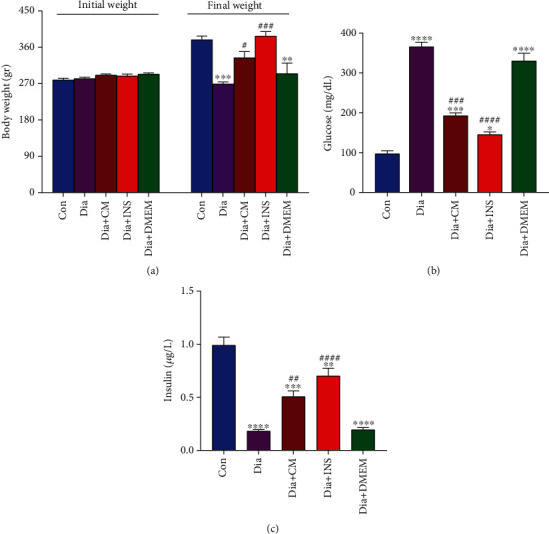
Body weight (a), blood glucose (b), and insulin (c) levels in the experimental groups. The values are presented as mean ± SEM (*n* = 7). Significant differences versus the control (^∗^*P* < 0.05, ^∗∗^*P* < 0.01, ^∗∗∗^*P* < 0.001, ^∗∗∗∗^*P* < 0.0001) and diabetes rats (^#^*P* < 0.05, ^##^*P* < 0.01, ^###^*P* < 0.001, ^####^*P* < 0.0001). Con: control; Dia: diabetes; Dia + CM: WJMSC-CM-treated diabetic group; Dia + INS: insulin-treated diabetic group; Dia + DMEM: DMEM-treated diabetic group.

The body weight of rats in five experimental groups was measured weekly. As shown in Figure 3, there was no difference in initial body weight among groups before diabetes induction and treatment. Significant weight loss was observed in untreated diabetic rats, and there is significant difference in final body weight between diabetic group and control. However, treatment with WJMSC-CM or insulin prevented weight loss and increased body weight in Dia + CM and Dia + INS compared to diabetic group (Figure 3(a)).

When compared to the control group, blood glucose (Figure 3(b)) and insulin (Figure 3(c)) levels in the diabetes group were significantly higher and lower, respectively (both, *P* < 0.001). Although diabetic rats treated with WJMSC-CM and insulin had significantly lower blood glucose levels (*P* < 0.001) and higher blood insulin levels (*P* = 0.0017) than untreated diabetic rats, they did not reach the levels of the control group. There were no significant differences in body weight, blood glucose, and insulin levels between the DMEM-treated and untreated diabetic groups.

### 3.2. Behavioral Test

#### 3.2.1. Morris Water Maze

**Figure 4:**
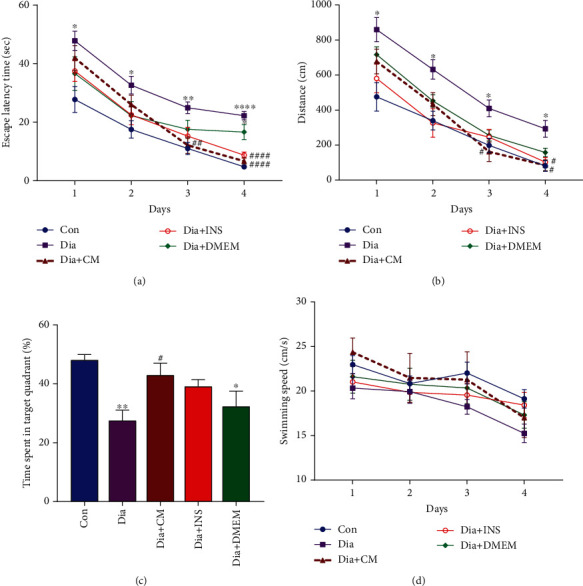
Effects of WJMSC-CM and insulin on spatial learning and memory in the Morris water maze in diabetic rats. The administration of WJMSC-CM improved the spatial learning performance in diabetic rats and significantly decreased the escape latency time (a) and traveled distance (b) on days 3 and 4. Using insulin improved spatial learning only on day 4 (two-way ANOVA). In the probe trial, after the removal of the platform, the percentage of target quadrant time (c) was significantly increased in Dia + CM group. The values are presented as mean ± SEM (*n* = 7). Significant differences versus the control (^∗^*P* < 0.05, ^∗∗^*P* < 0.01, ^∗∗∗^*P* < 0.001, ^∗∗∗∗^*P* < 0.0001) and diabetes rats (^#^*P* <0.05, ^##^*P* <0.01, ^####^*P* <0.0001). Con: control; Dia: diabetes; Dia + CM: WJMSC-CM-treated diabetic group; Dia + INS: insulin-treated diabetic group; Dia + DMEM: DMEM-treated diabetic group.

Animals' spatial learning and memory abilities were examined using the Morris water maze (MWM) test, one of the most widely used procedures in behavioral research. As shown in Figure 3, diabetes resulted in significantly (*P* < 0.05) higher escape latency time (Figure 4(a)) and traveled distance (Figure 4(b)) to find the platform in 4 days when compared to equivalent measures in the control group. On days 3 and 4, the Dia + CM group demonstrated a decrease in escape latency time and traveled distance compared to the diabetes group (both, *P* < 0.05). In addition, the Dia + INS group spent significantly less time finding the platform than the diabetes group, and the distance traveled to the hidden platform in the Dia + INS group was significantly (*P* < 0.05) less than the diabetes group only on day 4.

During the probe trial, the percentage of time spent in the target quadrant (Figure 4(c), *P* < 0.01) was significantly lower in the diabetes group compared to the control group. Contrarily, the administration of WJMSC-CM, but not insulin, resulted in a significant increase in the percentage of target quadrant time in the Dia + CM group (*P* < 0.05). Furthermore, there were no differences in swimming speed between the experimental groups (Figure 4(d)). The learning and memory ability did not differ between the Dia + DMEM and diabetes groups but were significant compared to controls.

#### 3.2.2. Open Field Test

**Figure 5:**
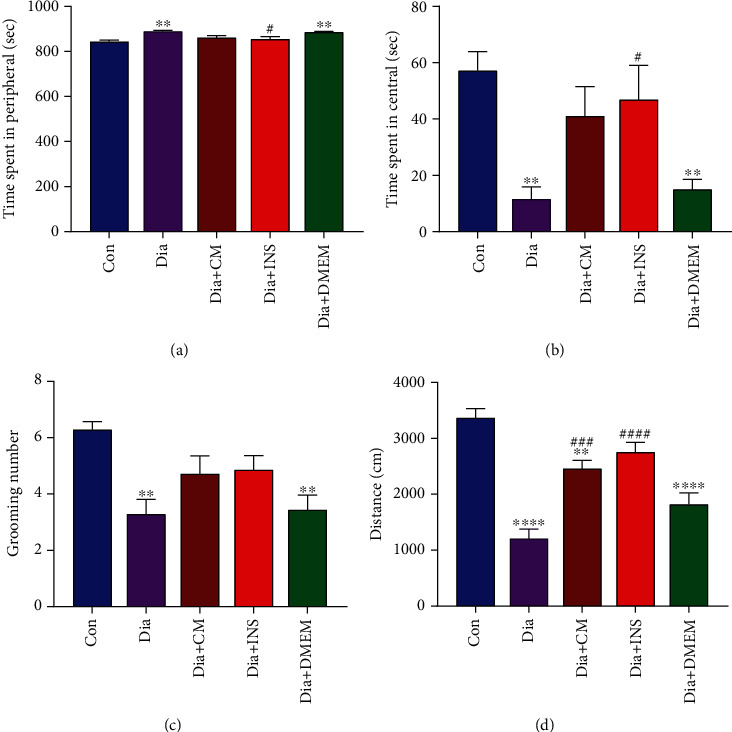
Effects of WJMSC-CM and insulin on anxiety-like behaviors in open field tasks in diabetic rats. Compared to the untreated diabetic group, one-way ANOVA analysis demonstrated a decrease in peripheral time (a) and an increase in central time (b) in WJMSC-CM- and insulin-treated diabetic rats, but only significant in the Dia + INS group. The grooming number was restored to the control level in both Dia + CM and Dia + INS groups (c). The administration of WJMSC-CM and insulin significantly increased the total distance traveled (d) in WJMSC-CM- and insulin-treated diabetic groups. The values are presented as mean ± SEM (*n* = 7). Significant differences versus the control (^∗∗^*P* < 0.01, ^∗∗∗∗^*P* < 0.0001) and diabetes rats (^#^*P* < 0.05, ^###^*P* < 0.001, ^####^*P* < 0.0001). Con: control; Dia: diabetes; Dia + CM: WJMSC-CM-treated diabetic group; Dia + INS: insulin-treated diabetic group; Dia + DMEM: DMEM-treated diabetic group.

The open field test (OFT) was applied to measure anxiety-like behaviors. As indicated in Figures 5(a)–5(d), compared to the control group, the time spent in the periphery significantly was increased (Figure 5(a), *P* < 0.0001), while time spent in the center (Figure 5(b), *P* < 0.0001), the number of grooming (Figure 5(c), *P* < 0.05), and total distance traveled (Figure 5(d), *P* < 0.0001) were significantly reduced in the diabetes group. Compared to the untreated diabetic group, however, WJMSC-CM partially recovered, and insulin significantly decreased the time spent in the periphery in treated diabetic groups (Figure 5(a)). In addition, the time spent in the center (Figure 5(b)) and the number of grooming (Figure 5(c)) in WJMSC-CM- and insulin-treated groups were higher than in the untreated diabetic group. Total distance traveled (Figure 5(d)) was significantly increased in Dia + CM and Dia + INS groups compared to the diabetes group. These OFT parameters were significantly different between the DMEM-treated group and the control group.

### 3.3. Oxidative Markers

**Figure 6:**
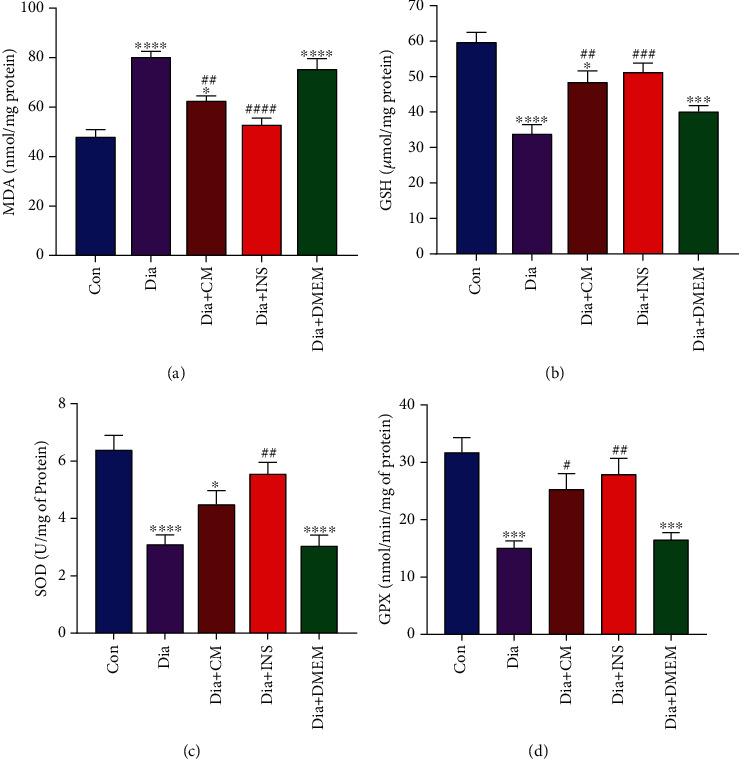
Effects of WJMSC-CM and insulin on oxidative stress markers in hippocampus of diabetic rats. According to one-way ANOVA (post hoc: Tukey) analysis, administration of WJMSC-CM improved the hippocampus levels of MDA and GSH, as well as the activity of GPx in diabetic rats. Insulin restores all oxidative stress parameters to normal value. The values are presented as mean ± SEM (*n* = 6). Significant differences versus the control (^∗^*P* < 0.05, ^∗∗∗^*P* < 0.001, ^∗∗∗∗^*P* < 0.0001) and diabetes rats (^#^*P* < 0.05, ^##^*P* < 0.01, ^###^*P* < 0.001, *^####^P* < 0.0001). Con: control; Dia: diabetes; Dia + CM: WJMSC-CM-treated diabetic group; Dia + INS: insulin-treated diabetic group; Dia + DMEM: DMEM-treated diabetic group.

The hippocampus levels of MDA and GSH, as well as the specific activities of SOD and GPx, are illustrated in Figures 6(a)–6(d). The MDA levels in diabetic rats were found to be significantly higher than in the control group (*P* < 0.001). The MDA concentration was significantly reduced in the Dia + CM (*P* < 0.01) and Dia + INS (*P* < 0.001) groups compared to the diabetes group (Figure 6(a)), but did not reach the control group in the Dia + CM group.

The diabetes group had significantly lower hippocampal GSH levels than the control group (*P* < 0.0001). Although treatment of diabetic rats with WJMSC-CM (*P* < 0.01) and insulin (*P* < 0.001) significantly increased hippocampal GSH concentration, it was significantly lower than the control group (Figure 6(b)).

As depicted in Figure 6(c), the diabetes group had significantly lower SOD antioxidant activity than the control group (*P* < 0.0001). Although WJMSC-CM therapy enhanced hippocampal SOD activity in diabetic rats, the difference was not significant when compared to the untreated diabetes group. In contrast, the SOD activity in the insulin-treated diabetic group was considerably higher than in the untreated diabetic group (*P* < 0.01).

The untreated diabetic group had significantly lower GPx specific activity than the control group (*P* < 0.001). In comparison to the untreated diabetic group, GPx activity was significantly elevated in the WJMSC-CM-treated group (*P* < 0.05) and insulin-treated group (*P* < 0.01) (Figure 6(d)).

In comparison to the untreated diabetic group, DMEM treatment did not affect all of the above oxidative measures. However, between the DMEM-treated group and the control group, there were considerable differences in hippocampus levels of MDA and GSH as well as antioxidant enzyme activity of SOD and GPx.

### 3.4. Gene Expression of Bax, Bcl-2, BDNF, and TNF-ɑ

**Figure 7:**
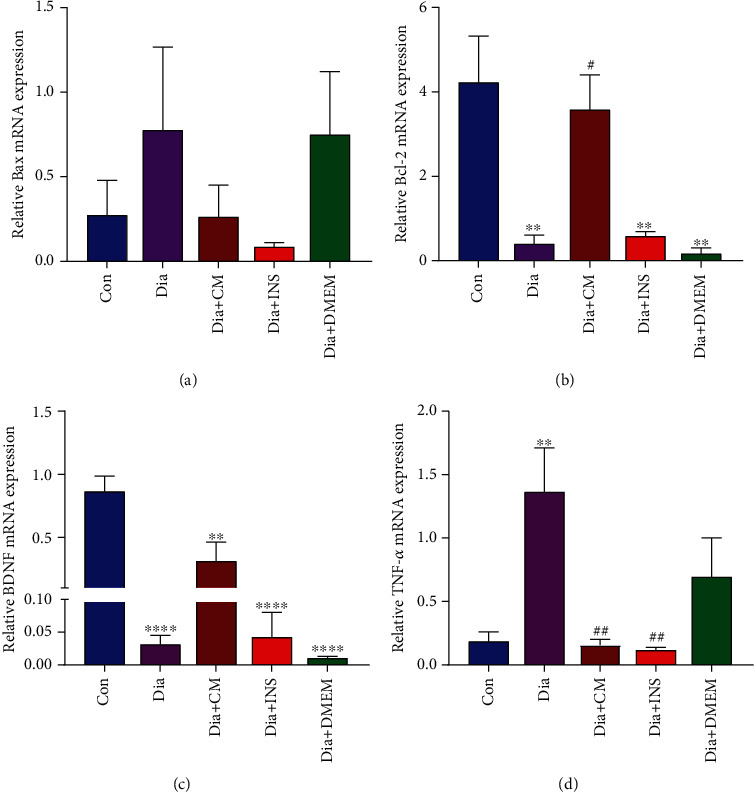
Effects of WJMSC-CM and insulin on Bax (a), Bcl-2 (b), BDNF (c), and TNF-*α* (d) gene expression levels in the hippocampus of diabetic rats. WJMSC-CM significantly increased and decreased hippocampal gene expression levels of Bcl-2 and TNF-*α* in diabetic rats, respectively. The values are presented as mean ± SEM (*n* = 5). Significant differences versus the control (^∗∗^*P* < 0.01, ^∗∗∗∗^*P* < 0.0001) and diabetes rats (^#^*P* < 0.05, ^##^*P* < 0.01). Con: control; Dia: diabetes; Dia + CM: WJMSC-CM-treated diabetic group; Dia + INS: insulin-treated diabetic group; Dia + DMEM: DMEM-treated diabetic group.

RT-PCR revealed that although Bax gene expression was higher in the diabetes group than in the control group, this difference was not statistically significant. Administration of WJMSC-CM and insulin for 60 days did not significantly reduce the mRNA expression level of Bax in Dia + CM and Dia + INS in comparison to the untreated diabetes group. There was no significant difference in the mRNA expression level of Bax between the Dia + DMEM group and the diabetes group (Figure 7(a)).

On the other hand, the mRNA expression level of Bcl-2 in the diabetes group was significantly lower than in the control group (*P* < 0.01). In the Dia + CM group, expression level of *Bcl-2* mRNA was significantly increased compared to the diabetes group (*P* < 0.05). There was no significant difference in Bcl-2 expression level among Dia + INS, Dia + DMEM, and diabetes groups (Figure 7(b)).

Also, the expression level of *BDNF* mRNA in the diabetes group was significantly decreased compared to the control group (*P* < 0.0001). In Dia + CM and Dia + INS groups, the mRNA expression level of BDNF was nonsignificantly higher than the diabetes group. There was no significant difference in the mRNA expression level of BDNF between the Dia + DMEM group and the diabetes group (Figure 7(c)).

As well, the mRNA expression level of TNF-ɑ in the diabetes group was significantly higher than the control group (*P* < 0.01). However, the administration of WJMSC-CM and insulin significantly decreased the expression level of *TNF-ɑ* mRNA compared to the untreated diabetes group (*P* < 0.01; Figure 7(d)).

DMEM treatment did not affect the gene expression level of Bax, Bcl-2, BDNF, and TNF-ɑ compared to the diabetes group. However, there were significant differences in hippocampus gene expression levels of Bax, Bcl-2, BDNF, and TNF-ɑ between the Dia + DMEM group and the control group.

### 3.5. Histomorphological Assessments of the Hippocampus

The majority of cells in the hippocampus are packed densely into a thin layer that curves into a double C-shaped structure. Based on the cytoarchitectural distinction of principle cells in the hippocampus, it was divided into three subdivisions as Cornu Ammonis 1 (CA1), Cornu Ammonis 3 (CA3), and dentate gyrus (DG). CA2 is simply a transition zone with a mixture of CA1 and CA3 cells.

**Figure 8:**
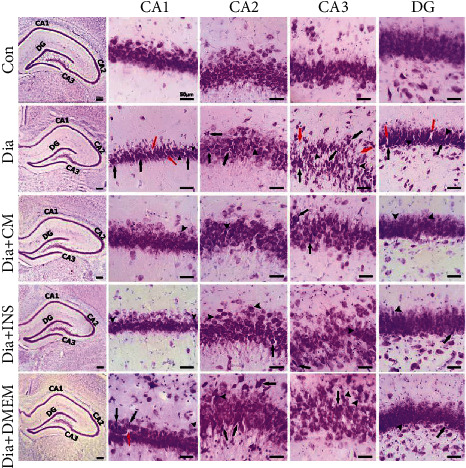
The representative cresyl violet-stained sections of the Cornu Ammonis (CA1, CA2, and CA3), and dentate gyrus (DG) regions in rat hippocampus of different groups. Darkened and contracted cells (black arrows), areas of cell loss (red arrows), and pyknotic nuclei (arrowheads). Con: control; Dia: diabetes; Dia + CM: WJMSC-CM-treated diabetic group; Dia + INS: insulin-treated diabetic group; Dia + DMEM: DMEM-treated diabetic group.

Light microscopic examination of cresyl violet-stained sections of all groups showed C-shaped hippocampus (Figure 8). In the control group (Figure 8, upper panel), CA1 is formed of 5–6 compact layers of small pyramidal cells arranged with a specific palisade appearance. The pyramidal cells indicated vesicular nuclei and pale basophilic cytoplasm. CA3 subregion is formed from a zone of large pyramidal cells, while area CA2 contains a mixture of large and small pyramidal cells. DG is formed of compact layers of small granule cells.

Examination of the untreated diabetes group (Figure 8, second panel) revealed marked disorganization, dispersion, and cell loss of small pyramidal cells in CA1 and CA2 regions darkened small pyramidal cells with a hyperchromatic pyknotic nucleus. Additionally, in the hippocampal CA3 region, there was cellular disorganization, increased intercellular spaces with many areas of cell loss, and shrinkage in the size of the large pyramidal cells, with dark pyknotic nuclei. The dentate gyrus also indicated marked disorganization. Many degenerated granule cells with pyknotic nuclei and areas of cell loss were also detected in the dentate gyrus.

After WJMSC-CM or insulin treatment, the above-mentioned cell death was significantly improved. Cresyl violet-stained sections of the diabetic group that received WJMSC-CM (Figure 8, third panel) showed preservation of the smallest pyramidal cells of CA1 and CA2 regions. Most of them showed vesicular nuclei, and few nuclei were pyknotic. Large pyramidal cells of the CA3 region were preserved. Most of them had centrally located vesicular nuclei except for a few apoptotic cells with pyknotic nuclei. The dentate gyrus was preserved, and the granule cells were densely arranged.

Insulin therapy also caused a significant improvement in the preservation of the pyramidal and granular cells with less marked shrinkage and few apoptotic cells with pyknotic nuclei (Figure 8, 4th panel).

In the DMEM-treated group (Figure 8, lowest panel) similar to the untreated diabetes group, marked cellular disorganization, dispersion, many apoptotic pyramidal cells with pyknotic nuclei, and cell loss of small pyramidal cells were observed in CA1 and CA2 regions. In addition, in the CA3 and dentate gyrus regions, there were contracted cells with pyknotic nuclei.

Based on the morphological observation of the rat hippocampus, the cognitive function of diabetic rats may be related to the morphological changes of the hippocampal neurons, and WJMSC-CM could significantly alleviate hippocampal neuronal damage caused by diabetes.

### 3.6. Hippocampus Volume Estimation

**Figure 9:**
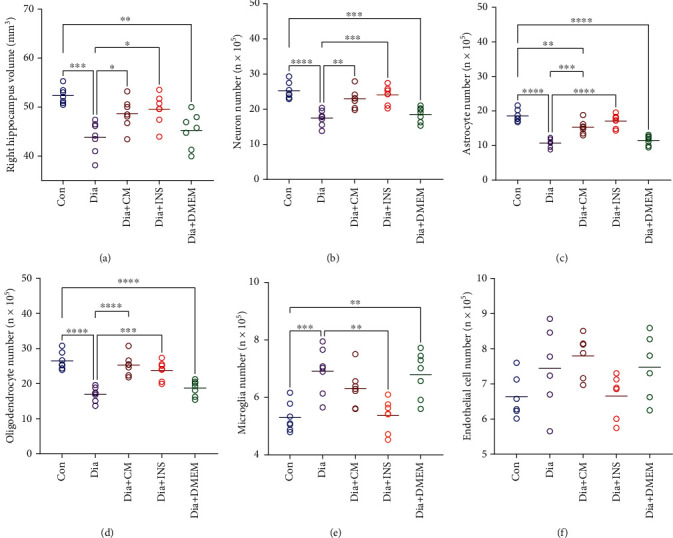
Effects of WJMSC-CM and insulin on the volume (a) and numbers of neurons (b), astrocytes (c), oligodendrocytes (d), microglia (e), and endothelial cells (f) of the hippocampus. The values are presented as mean ± SEM (*n* = 7). ^∗^*P* < 0.05, ^∗∗^*P* < 0.01, ^∗∗∗^*P* < 0.001, and ^∗∗∗∗^*P* < 0.0001. Con: control; Dia: diabetes; Dia + CM: WJMSC-CM-treated diabetic group; Dia + INS: insulin-treated diabetic group; Dia + DMEM: DMEM-treated diabetic group.

The total volume of the right hippocampus in the diabetes group was significantly decreased when compared to the control group (*P* < 0.001). On the other hand, in the WJMSC-CM treated group and insulin-treated group, the total volume of the right hippocampus was significantly higher than in the untreated diabetes group (*P* < 0.05). There was no significant difference in hippocampus volume between the DMEM-treated group and the diabetes group (Figure 9(a)).

### 3.7. Number of Neurons

Counting the cresyl violet stained neurons of the hippocampus showed a highly significant decrease in the total number of neurons in the diabetes group (*P* < 0.0001) in comparison to the control group. However, the administration of WJMSC-CM (*P* < 0.01) or insulin (*P* < 0.001) successfully reversed this diabetes-induced reduction in the hippocampal regions. No difference was found in the total number of neurons between the DMEM-treated group and the diabetes group. (Figure 9(b)).

### 3.8. Number of Glial Cell Types

#### 3.8.1. Number of Astrocytes

In the hippocampus of the diabetes group, the number of astrocytes was significantly decreased compared to the control group (*P* < 0.0001). Diabetic rats that received WJMSC-CM (*P* < 0.001) or insulin (*P* < 0.0001) exhibited a significant increase in the number of astrocytes; however, the DMEM-treated group did not show a prominent effect on this parameter (Figure 9(c)).

#### 3.8.2. Number of Oligodendrocytes

The diabetes group showed a significant reduction in the number of oligodendrocytes in the hippocampus in comparison to the control group (*P* < 0.0001). However, treatment with WJMSC-CM (*P* < 0.0001) or insulin (*P* < 0.001) significantly increased oligodendrocyte numbers compared to the untreated diabetes group. No significant difference was observed between the DMEM-treated group and the diabetes group (Figure 9(d)).

#### 3.8.3. Number of Microglia

Unlike other glial cells, the number of microglia in the hippocampus of the diabetes group was significantly higher than the control group (*P* < 0.001). Diabetic rats treated with insulin revealed a significant reduction in the number of these cells (*P* < 0.01); however, other treatments did not have a marked effect on microglia numbers (Figure 9(e)).

#### 3.8.4. Number of Endothelial Cells

Our data showed that the number of endothelial cells slightly increased in the WJMSC-CM-treated group, but no statistically significant differences were found among the groups (Figure 9(f)).

## 4. Discussion

In the current study, we demonstrated the therapeutic potential of WJMSC-CM on cognitive and memory impairment in the STZ-induced rat model of diabetes, which was associated with ameliorated hyperglycemia, improved spatial learning and memory tasks, attenuated oxidative stress, and reduced inflammation and apoptosis in hippocampal tissue.

The present investigation first confirmed that STZ caused sustained hyperglycemia as a result of reduced insulin secretion. The hippocampus, an important integration center for learning and memory, is sensitive to changes in insulin and glucose concentrations and is well known to have densely distributed insulin receptors [39]. Cognitive dysfunction and impaired synaptic plasticity in both types of diabetes have been associated with hyperglycemia, insulin deficiency, and/or insulin resistance and altered insulin signaling [40, 41]. The neuronal loss in the hippocampus as well as memory impairment have been confirmed in numerous animal models of diabetes [42]. Therefore, the progression of neuronal degeneration in the hippocampus of diabetic rats might be effectively prevented by controlling blood glucose levels. Our results revealed that diabetic animals treated with WJMSC-CM for 60 days improved hyperglycemia and increased insulin secretion in treated diabetic rats. Furthermore, a study by Nugroho et al. indicated that treatment with stem cell-derived conditioned medium in diabetic rat not only regenerate pancreatic *β* cell but also maintain their function to produce insulin [43].

Consistent with previous research, our findings demonstrated that learning and memory abilities were impaired by diabetes. In the Morris water maze (MWM) task, four days of learning performances including escape latency time and traveled distance were increased, while time spent in the target quadrant decreased in STZ-induced diabetic rats. These results agree with other findings indicating that diabetes leads to impaired spatial memory and cognition deficits [44–46].

In the open field test, this work confirms that diabetes mellitus plays a role in the development of anxiety-like behaviors. In line with the other findings [47, 48], STZ caused a significant increase in anxiety-like behaviors which were shown with a decrease in time spent in the central part, total distance traveled, and the number of grooming when compared to the control group. While the WJMSC-CM and insulin-treated diabetic groups were associated with a reduction in anxiety-like behaviors as evidenced by an increase in time spent in the central part and total distance traveled when compared with the untreated diabetic group. We found a nonsignificant increase in the number of grooming in the WJMSC-CM and insulin-treated diabetic groups. However, previous studies indicated that the grooming number alone might not be a reliable indicator of animal stress and anxiety, and it could be interpreted as decision-making and displacement behavior [48–50]. Furthermore, the MWM task and open field test showed that administration of WJMSC-CM and insulin significantly increased learning and memory capacity and attenuated anxiety-like behaviors.

Increased free radical and lipid peroxidation is a well-known mechanism related to neuronal damage [51]. Multiple mechanisms, including oxidative stress, antioxidant enzyme suppression, inflammation, and apoptosis have been hypothesized to account for the deleterious effects of diabetes-induced hyperglycemia on the hippocampus [52]. The findings of this study showed that in the hippocampus of diabetes rats, the MDA level (as a lipid peroxidation marker) was significantly elevated, whereas GSH content and enzyme activity of GPx and SOD were considerably lowered. It has previously been manifested that in STZ-induced diabetic rats, MDA and antioxidative enzymes including catalase, glutathione, and superoxide dismutase are changed not only in plasma but also in other organs including the brain [53]. Our study showed that treatment with WJMSC-CM and insulin recover the levels of MDA, GSH, GPx, and SOD nearly to control values. Some researchers also reported that mesenchymal stem cells (MSCs) or their secretory vesicles, called exosomes, can prevent hippocampal neurons from oxidative stress [54, 55]. Bodart-Santos et al. showed that human Wharton's jelly mesenchymal stem cell extracellular vesicles protect hippocampus neurons from amyloid beta oligomers induced oxidative stress [56]. Several studies indicated that exosomes in condition media are neuroprotective against oxidative stress in aging brain and Alzheimer's disease [57, 58]. Also, amyloid-oligomer-induced oxidative stress and synapse damage to hippocampal neurons are prevented by mesenchymal stem cell-derived extracellular vesicles [55].

With proteomic analysis, researchers confirmed that WJMSCs release a high amount of HGF and other important neurotrophic factors including fibroblast growth factor-2 (FGF2), brain-derived neurotrophic factor (BDNF), and basic nerve growth factor (bNGF), and also angiogenesis factor such as vascular endothelial growth factor A (VEGFA) [59, 60]. Some of these neurotrophic factors such as HGF and FGF can reduce ROS in tissues or organs [61, 62]. Thus, WJMSC-CM may protect against diabetes-associated memory decline by reducing oxidative stress.

It has been noted that hyperglycemia-related oxidative stress contributes to the activation of major proinflammatory signaling pathways [63], and diabetes could lead to massive neuroinflammation and the consequent increased microglial activation [3, 64, 65]. Microglial activation was well known to induce neuron damage via increasing cytokines and reactive oxygen species (ROS), thus inducing neurotoxicity [66–68]. Moreover, microglial activation and production of proinflammatory cytokines and neurotoxic mediators, including interleukin-1 beta (IL-1*β*), tumor necrosis factor-alpha (TNF-ɑ), nitric oxide (NO), and ROS, can result in neuronal loss and cognitive deficits [42, 69]. The cytokine TNF-*α* is a key controller of neuroinflammation associated with many neurodegenerative diseases, and overexpression of TNF-*α* in the hippocampus impairs memory and synaptic plasticity [42]. The current study showed elevated hippocampal TNF-*α* expression in diabetic rats. Surprisingly, WJMSC-CM and insulin were able to reverse this trend, thereby displaying anti-inflammatory actions. An in vitro investigation demonstrated that MSC-conditioned media reduced the expression of proinflammatory factors such as IL-1, TNF-*α*, and IL-6 in astrocytes [70]. Thus, suppression of TNF-*α* expression in the hippocampus by WJMSC-derived secretory factors may prevent neuronal loss, stimulate synaptogenesis, and subsequently sustain cognitive function. On the other hand, we found that in the hippocampus of STZ-induced diabetic rats, proapoptotic factor Bax was increased while antiapoptotic factor Bcl-2 was decreased. The Bcl-2 family proteins play a key role in the regulation and control of apoptosis and are particularly critical for mitochondrial pathway-mediated apoptosis. A key factor in determining apoptosis is the ratio of the antiapoptotic protein Bcl-2 to the apoptotic protein Bax, which can ultimately initiate the caspase cleavage cascade [71, 72]. It has been indicated that diabetes induces neuronal apoptosis by downregulating antiapoptotic Bcl-2 expression and upregulating the proapoptotic Bax gene [73]. Our study exhibited that WJMSC-CM can reduce diabetes-induced neuronal apoptosis in the hippocampus of rats. Huang et al. reported that mesenchymal stem cell-conditioned medium prevents radiation damage to hippocampal neurons in animal models with Alzheimer's disease by reducing oxidative stress and apoptosis [74]. BDNF is primarily expressed in the central nervous system and plays a key role in neurogenesis [75, 76]. Changes in the levels of various neurological factors, including brain-derived neurotrophic factor (BDNF), nerve growth factor (NGF), and glial cell-derived neurotrophic factor (GDNF), have been linked to cognitive deficiencies [77]. WJMSC-CM, on the other hand, contain a range of neuromodulators that are beneficial to neuronal survival and neurogenesis. The current study indicated that WJMSC-CM could alleviate the decrease of BDNF caused by STZ-induced diabetes. This effect might be due to the accumulation of WJMSC-CM-derived BDNF in the brain tissue or increased BDNF secretion that was mediated by other cytokines derived from WJMSC-CM.

Afterward, we conducted further histological and stereological evaluations in the hippocampus to examine the effects of WJMSC-CM and insulin treatments at the cellular level. The histological findings of present study showed that 60 days of diabetes caused hippocampal damage, which was followed by a decrease in the hippocampal volume and neuronal population. Hyperglycemia induced by injecting STZ also reduced the number of hippocampal astrocytes and oligodendrocytes and increased the number of hippocampal microglia. Obtained stereological findings are consistent with the previous reports displaying diabetes induced apoptosis and severe neuronal loss in the hippocampus of rodents [78–80]. Other researchers have found neuronal cell death in the hippocampus of STZ-induced diabetic rats after 21 days following STZ injection [81]. In a study, accountable histological alterations were observed in various subregions of the hippocampus (CA1, CA2, CA3, dentate hilus, and dentate gyrus) in 45 and 60 days of diabetes [82]. Zhou et al. reported neuronal cell death, especially at the CA3 and dentate gyrus regions of the hippocampus in STZ-induced diabetic rats [83]. Therefore, we speculated that a decline in the number of neurons is a crucial factor in cognitive dysfunction associated with diabetes, and it is suggested that the decrease in hippocampus volume was induced by the degeneration of glial cells, blood vessels, nerve fibers, etc.

Various growth-promoting factors such as insulin-like growth factor-I (IGF-I), HGF, bFGF, VEGF, NGF, and BDNF were identified in the WJMSC-derived CM that exert a neuroprotective effect on damaged neurons [84]. Several studies showed that hippocampal neurogenesis can be influenced by IGF-I [85], NGF [86], VEGF, FGF-2, and BDNF [87]. Additionally, the primary impact of WJMSC-derived CM on the brain is also supported by evidence that explains many routes by which growth factors might cross the blood-brain barrier (BBB). IGF-1 can pass the BBB via transcytosis pathways mediated by receptors [88]. Basic FGF is delivered from the blood to the brain via adsorptive transcytosis [89], whereas FGF21 crosses the BBB via simple diffusion [90]. By using receptor-mediated transport, NGF can pass through the blood-brain barrier [91]. The intact BDNF in the peripheral circulation can cross the BBB via a highly saturated and capacity transport route [92]. VEGF increases BBB endocytosis and transcytosis and can allow transport across the BBB [93]. Furthermore, extracellular vesicles, or exosomes, in condition medium that released by MSCs are paracrine mediators that promote tissue regeneration by encapsulating and delivering active biomolecules including various peptides, proteins, and RNA species to injured cells/tissues [94]. In addition, the control of blood glucose levels in diabetic rats treated with insulin or WJMSC-CM could effectively prevent the progression of neuronal damage in the hippocampus, as evidenced by the experimental results showing that the number of apoptotic cells in the diabetic group receiving treatment was significantly lower than that of the untreated diabetic group.

Findings from several studies have demonstrated that insulin besides being essential in the regulation of glucose homeostasis exerts neurotrophic and neuromodulatory effects in a variety of central and peripheral neuronal systems [95], and insulin signaling plays a key role in neuronal survival [96] and learning and memory [41]. In addition, insulin regulates mitochondrial metabolism and oxidative capacity [97, 98]. Insulin also showed an anti-inflammatory effect through modulating proinflammatory signaling pathways [63, 99], reducing oxidative stress and neuroinflammatory response as well as the production of cytokines, such as IL-1*β* and TNF-*α* [100].

## 5. Conclusion

Our present results demonstrated that WJMSC-CM and insulin produced improving effects on metabolic and behavioral alterations as well as biochemical and histopathological outcomes in the hippocampus. The present study showed that WJMSC-CM ameliorated diabetes-induced cognitive decline in rats, at the levels of oxidative stress and peripheral inflammation associated with the development of cognitive impairment. It also successfully prevented hippocampal neuronal and nonneuronal cell death in diabetic rats, which suggested the therapeutic potential of WJMSC-CM in diabetes-induced learning and memory impairment. The protective effects of WJMSC-conditioned medium could be attributed to high levels of active growth factors and proteins produced by mesenchymal stem cells, including BDNF, NGF, VEGF, and bFGF and their capacity to reduce oxidative damage.

This finding may provide insight into the practical application of WJMSC-CM in the treatment of diabetes complications, including cognitive impairment. However, further research is required to identify the exact mechanisms, efficacy, and safety of different dosages and injection times before using WJMSC-CM in a clinical trial.

## Figures and Tables

**Table 1:** Bax, Bcl-2, BDNF, TNF-*α*, and GAPDH primer sequences used for RT-PCR reaction.

Primer	Forward	Reverse	Amplicon length
Bax	GCTACAGGGTTTCATCCAGG	TTGTTGTCCAGTTCATCGCC	141
Bcl-2	CTGGTGGACAACATCGCTCT	GCATGCTGGGGCCATATAGT	115
BDNF	AAGCCGAACTTCTCACAT	TGGTCATCACTCTTCTCAC	138
TNF-ɑ	TCAGCCTCTTCTCATTCCTGC	TTGGTGGTTTGCTACGACGTG	203
GAPDH	AGTGCCAGCCTCGTCTCATA	GAGAAGGCAGCCCTGGTAAC	91

## Data Availability

The experimental data used to support the findings of this study are available from the corresponding author upon request.
